# Development and implementation of the ECHO model in a school setting to address youth electronic cigarette use in Kansas: A protocol

**DOI:** 10.3389/fpubh.2022.1057600

**Published:** 2023-01-12

**Authors:** Eleanor L. S. Leavens, Jordan Roberts, Babalola Faseru, Mark Thompson, Karin Denes-Collar, Hina Shah

**Affiliations:** ^1^Department of Population Health, School of Medicine, University of Kansas, Kansas City, KS, United States; ^2^University of Kansas Cancer Center, Kansas City, KS, United States; ^3^Kansas Department of Health and Environment, Topeka, KS, United States; ^4^Kansas State Department of Education, Topeka, KS, United States; ^5^Masonic Cancer Alliance, The University of Kansas Cancer Center, Kansas City, KS, United States; ^6^Kansas Health Institute, Topeka, KS, United States

**Keywords:** tobacco, electronic cigarette, school, prevention, cessation, ECHO

## Abstract

**Introduction:**

Schools remain at the frontlines of addressing issues, such as e-cigarette use, that impact students. Despite e-cigarette use remaining a significant public health concern in the U.S., schools have limited resources (e.g., staff, capacity, programming) to address it, especially in rural and frontier areas. This ECHO Pilot Project aimed to build capacity and equip schools and school staff in the state of Kansas to address high rates of youth e-cigarette use by providing prevention support and information on best practices for e-cigarette cessation.

**Methods and analysis:**

The pilot used the established Project ECHO model to disseminate evidence-based strategies for e-cigarette prevention and cessation among youth to schools across Kansas. The pilot selected 20 interdisciplinary school teams representing both rural and urban middle and high schools across the state to participate in seven ECHO sessions. ECHO sessions proceeded throughout Fall 2021, with the final session in Spring 2022. School participants completed pre-post surveys as well as component-specific surveys following each ECHO session. In addition, each school team created an individualized action plan to comprehensively address e-cigarette use at their school based on the information provided throughout the ECHO. Survey data, school tobacco/nicotine policies, and action plans will be analyzed to assess process and final outcomes.

**Discussion:**

If successful, this pilot will demonstrate that the ECHO model is an effective platform for building school staff knowledge and skills to implement evidence-based strategies in both urban and rural settings. It is anticipated that the pilot will build capacity and equip schools and school staff to address high rates of youth e-cigarette use by providing support for school-based prevention programs and referrals for e-cigarette cessation which will lessen the burden of nicotine-related problems in Kansas schools and communities. Finally, the pilot will provide evidence that the ECHO model can be successfully and equitably applied in a school setting and may be a viable method for addressing other public health-related issues faced by schools.

## Introduction

Electronic cigarettes (e-cigarettes) are battery-powered devices that heat and aerosolize nicotine-containing liquid which is inhaled and delivered to the user. While e-cigarettes are likely less harmful than combustible cigarettes (1), they are not without risk (2), particularly for youth and individuals that are otherwise naïve to tobacco (3, 4). For this reason, use of e-cigarettes among youth remains a significant public health concern. National data suggest that approximately 11% of high school students and 3% of middle school students report current (past 30-day) e-cigarette use (5) and in 2019, nearly a quarter (22.0%) of Kansas high school students reported current e-cigarette use (6). While overall use rates are plateauing or decreasing nationally, current users report more frequent use. Specifically, national surveys suggest that approximately 39% of high school and middle school e-cigarette users report e-cigarette use on at least 20 of the past 30 days (5), likely resulting in significant levels of nicotine dependence.

Schools play a critical role in promoting student health and are uniquely positioned to implement evidence-based tobacco prevention and cessation support for students. Evidence suggests that school-based prevention programs created to address youth tobacco use have proven to be effective in reducing initiation in the short term, and in some cases, achieve longer term cessation outcomes when programs implement sustainable policy, systems, and environmental changes (7). However, few comprehensive programs or resources are available to schools to implement such programs and address e-cigarette use, particularly in rural and frontier areas. For this reason, schools largely address e-cigarette use in a similar manner to other disciplinary issues. A study conducted by the Public Health Law Center that examined Kansas school disciplinary policies for e-cigarette use found that over 75% of middle and high schools used suspension at a first tobacco/nicotine offense while less than a quarter included offers of tobacco education or cessation support at a first offense (8). Unfortunately, discipline or even education alone have not been shown to be efficacious in achieving cessation (9). In fact, studies show that students who are suspended even once, are 44% less likely to graduate and almost 50% less likely to go on to college, highlighting the importance of implementing restorative practices in schools (10).

Nearly half (45.8%) of Kansas middle and high school students who currently use tobacco products tried to quit in the past year (6) and national data suggest that approximately 4% of youth e-cigarette users have experienced an unsuccessful quit attempt (11), supporting the need for comprehensive cessation support in schools. By equipping school staff with best practice information on prevention and intervention of e-cigarette use, students will have the support and resources necessary to increase the likelihood of cessation. However, because youth e-cigarette use is an emerging public health concern, there is limited data and best-practice recommendations specific to these products. Tobacco control programs that include a range of complementary and coordinated strategies such as school-based tobacco prevention programs can reduce prevalence of tobacco use among school-aged youth.

The Extension for Community Healthcare Outcomes (ECHO) Project is a model for global dissemination of expert-level education to medical providers and patients (12, 13). In recent years, and with COVID-19 as a catalyst, the ECHO model has been used to broadly disseminate information to educators and school personnel. The current pilot initiative aimed to build capacity and equip schools and school staff to address high rates of youth e-cigarette use by providing support for school-based prevention programs and referrals for e-cigarette cessation.

The goal of this pilot program was to apply the Whole School, Whole Community, Whole Child Model (WSCC) (14) and use a collaborative blended learning approach to equip critical personnel with e-cigarette cessation resources, tools, and techniques. The WSCC model is a student-centered framework developed by the Centers for Disease Control and Prevention for addressing health in schools, which calls for greater alignment between the public health and education sectors and emphasizes the importance of evidence-based school policies and practices. The WSCC model emphasizes the importance of building a network of community support around schools and youth to improve student health. The Kansas State Department of Education (KSDE) has applied the WSCC model to several initiatives to improve student learning and health, including work undertaken by Child Nutrition and Wellness, Social-Emotional Learning, and Health and Physical Education curriculum guidance, among others. This model and resulting work was consistent with KSDE's vision, “Kansas leads the world in the success of each student.” As a pioneer in the field, the pilot program aims: (1) To develop a network of stakeholders to help design, tailor, implement and evaluate strategies and interventions to reduce vaping in schools. (2) To build capacity to increase the confidence of schools and school personnel to address e-cigarette cessation. (3) To develop skills of school health professionals to provide support and referrals for e-cigarette cessation. (4) To evaluate the quality, effectiveness, and impact of the program within Kansas schools.

## Methods

### Pilot program description

The pilot program was a fully online experience. The pilot program included four structured learning components: (1) a summit to kick off the program, understand the issue, and share experiences; (2) a toolkit to develop and strengthen participants' knowledge and skills and provide resources for implementation in a school setting; (3) a specialized training experience based on the tele-mentoring approach of Project ECHO focused on implementing tobacco and nicotine prevention and cessation practices in middle and high schools in Kansas and (4) an action plan developed by each team to address e-cigarette use in their school.

### Partners

The pilot leveraged longstanding relationships with Kansas State Department of Education, the Kansas Department of Health and Environment, the University of Kansas Medical Center, the University of Kansas Cancer Center, Children's Mercy Kansas City, the Kansas Health Institute, Kansas Association of School Boards, Kansas Nursing Association, DCCCA, Kansas Attorney General's office, and youth programs such as Kansas RESIST, and other stakeholders to reduce e-cigarette use in middle and high schools.

### Target audience

School teams included up to five members and were comprised of teachers, school nurses, counselors, social workers, athletic trainers, health teachers, coaches, administrators, and student resource officers. This target audience was selected because of their role in the school and their potential influence among students.

### Recruitment and application

The ECHO hub team elicited applications from middle and high schools statewide with the goal of selecting 20 schools with two representing each of the 10 Kansas State Board of Education (KBOE) members' districts and a total of approximately 100 participants. A total of 49 schools submitted completed applications to participate in the pilot. Not all the KBOE districts were represented by the applicants, though the applicants did represent a geographical spread throughout the state.

#### Selection criteria

Selection criteria were designed to enroll schools and school teams that were representative of Kansas students. Specific criteria included diversity of geography between schools, rural/urban setting, socioeconomic status (SES; percent free and reduced lunch), and racial and ethnic make-up of the student body. In addition, pilot school applicants were evaluated on their motivation to impact student e-cigarette use as evidenced by previous efforts taken toward e-cigarette prevention and/or intervention. Applicants were also asked to assure their willingness to fully participate in the Project ECHO sessions. This assurance was listed as a pre-requisite for applying. Further, applicants were asked to show evidence of a group of stakeholders who would form a comprehensive team that, at a minimum, included a school health champion (e.g., school nurse, counselor) and a school administrator. To accomplish this, the application included a requirement that schools list their team members and their role. The application is included in the Supplementary Figure 1.

#### Selected schools

A total of 20 interdisciplinary school teams were selected, representing 21 schools. One team consisted of staff, including administrators, from two high schools in the same town. Of the 20 school teams, four were classified as urban, four were semi-urban, five densely settled rural, four rural, and three frontier. Eleven were high schools, five were junior/senior high schools, and four were middle schools. In terms of district size classification (1A being the smallest and 6A being the largest), two were 1A, four were 2A, four were 3A, two were 4A, five were 5A, and four were 6A.

### Deliverables

School teams were required to submit an individualized action plan at the end of the ECHO. Schools were instructed to use a SMART (specific, measurable, achievable, realistic, timely) goal approach when creating their action plans. Under this general model, action plans included a vision, goals, objectives, accountability log, and timeline. School teams were also provided an example action plan for reference and office hours for pre-submission feedback and brainstorming with a hub team member. The action plan instructions and template are included in the Supplementary Figure 2.

### Procedures

Table 1 includes an outline of specific topics covered during orientation, summit, and core ECHO sessions.

**Table 1:** Session overviews and content.

**Session**	**Presenter(s)**	Session content^*^
Orientation	ECHO hub team	School team introductions
		ECHO hub team introductions
		Overview of the program and ECHO model (use of technology to leverage scarce resources, share best practices to reduce disparities, employ case-based learning, monitor outcomes)
		Program timeline and deadlines for deliverables
		Expectations of school and hub teams
		Mock ECHO session by hub team to model future sessions
		Overview of case-based learning and case discussion (information needed to submit a case)
		Logistical overview (how to submit cases, how to access ECHO content and session video uploads)
		Action plan discussion (components, timeline)
		Questions and discussion with the entire group
Summit	(1) Medical Officer, Office on Smoking and Health, CDC (2) ECHO hub team	Keynote speaker from CDC
		Public Health Law Center presentation (“Equitable Enforcement of Vaping Policy in Kansas High Schools”)
		Panel session with school nurse, pediatrician, and district administrator
		Toolkit overview
		Questions and discussion with the entire group
ECHO 1: Tobacco prevention in the school setting	(1) School district director of health services (2) School district counseling and college and career readiness coordinator	Benefits of a tobacco-free school
		Three levels of prevention with focus on primary prevention in schools
		Steps to address vaping in schools – Policy implementation and enforcement – Establish student education plan – Engage students in the prevention effort – Train school staff – Provide parent e-cigarette education resources
		Questions and discussion with the entire group
ECHO 2: School-related legal issues around e-cigarette use	Assistant Executive Director for Legal Services (state school board) and Attorney	4^th^ amendment protections against unreasonable search and seizure
		Law enforcement standard
		Federal tobacco laws
		State level indoor clean air laws
		Use, purchasing, and distribution of vapes among minors
		Legal definitions of e-cigarettes
		Considerations for cannabis
		Alternatives to punishment
		Cessation education vs. treatment in the school setting
		Reporting of potentially criminal acts in schools
		Questions and discussion with the entire group
ECHO 3: Introduction to nicotine dependence and cessation	Clinical Psychologist and Assistant Professor	Background on nicotine dependence
		Cycle of nicotine dependence and withdrawal
		Evidence-based cessation intervention
		Overview of nicotine replacement therapy
		Ask-Advice-Connect with example language
		Cessation resources (community-based, one-on-one adult led programs, online/text-based programs, parent/caregiver support)
		Questions and discussion with the entire group
ECHO 4: Discussing cessation with students using a Motivational Interviewing approach	Psychologist and Professor	Overview of the standard approach to addressing health behaviors
		Principles of psychological reactance
		Scientific evidence for Motivational Interviewing
		Motivational Interviewing principles – Collaboration – Evocation – Autonomy-supporting – Empathy
		Putting motivational interviewing principles into practice with sample language – Seek permission and set agenda – Join up – Global summaries – Behavioral action plan
		Overview of general Motivational Interviewing skills for all conversations – Elicit-provide-elicit – Open-ended questions – Reflective listening
		Markers of successful Motivational Interviewing-consistent encounter
		Questions and discussion with the entire group
ECHO 5: Student-centered approach	(1) Executive Director, State high school activities organization (2) School Counselor (3) District Resource Officer	Panel discussion content: – Extracurricular involvement after e-cigarette policy violation – Bona Fide Student regulations – Relationship of social-emotional and mental health with e-cigarette use – Treatment of minoritized students and equity issues – Role of the student resource officer in addressing e-cigarette use
		Case presentation and discussion with entire group
		Overall questions and discussion with the entire group

* Session content includes information from didactic portions of the ECHO session. Each didactic portion was followed by a small group breakout case-based discussion.

#### Orientation

Every school team member was required to attend one of two orientation sessions. The objectives of the orientation were to share (1) the uniqueness of the ECHO, (2) the importance of the ECHO, and (3) the overarching and procedural goals of the ECHO with all participants and other stakeholders. The orientation lasted 1 h and 20 min and covered the timeline and expectations (e.g., expectation for participation by all individual team members, utilization of the toolkit, and responsiveness of the ECHO hub team) for the current ECHO program, background and origin of the broad ECHO model, tailoring of the current pilot to address e-cigarette use, procedures, timeline, and expectations (e.g., ECHO sessions, deliverables, assessments). During the orientation, the hub team introduced the topics for the five core ECHO sessions and corresponding content experts. Throughout the orientation, a strong emphasis was placed on the use of case-based learning. The hub team modeled case presentation and discussion.

#### Summit

Each school team attended a 3-h kickoff summit that aimed to provide a basic understanding of e-cigarettes, nicotine dependence, harms of e-cigarette use among youth, an overview of available resources, current challenges experienced by schools in addressing student health issues, and legal challenges in addressing e-cigarette use in schools. In addition, the summit included a keynote address and panel discussion. During the panel, an associate superintendent, pediatrician, and school nurse discussed challenges and barriers to addressing e-cigarette use in schools and strategies for success in implementing prevention and cessation strategies.

#### Toolkit

A web-based toolkit housed resources for implementation of e-cigarette prevention and cessation in the school setting. Toolkit chapters corresponded directly with the core ECHO sessions and were released the week preceding the ECHO session. Toolkit chapters included a brief introduction to the topic, resources for implementation of strategies in a school setting, and an introductory video. Toolkit chapters were developed in collaboration with a team of content experts who provided recommendations for content, feedback on content, and final approval.

#### Core ECHO sessions

The core ECHO sessions consisted of five learning sessions with specialized training using the ECHO model and two additional sessions dedicated for sharing action plans and implementation progress. The five specialized training sessions covered (1) tobacco prevention in the school setting, (2) school-related legal issues around e-cigarette use, (3) introduction to nicotine dependence and cessation, (4) discussing cessation with students using a Motivational Interviewing approach, and (5) using a student-centered approach. Consistent with the ECHO model, each training session began with a didactic and question/answer period followed by a case presentation and discussion. Didactics were provided to the whole group and case presentations and discussions were conducted in breakout rooms with 4–5 school teams, a trained facilitator, and a content expert. School teams were grouped in breakout rooms based on geographic area due to the likelihood of access to shared resources. The breakout room groups remained consistent each session with only the content expert changing based on the topic.

### Pilot evaluation

An evaluation of the pilot was designed to understand how well the program worked to achieve the intended goals, to improve its effectiveness, and to inform programming decisions. A logic model that guided program development and will guide evaluation is presented in Table 2.

**Table 2:** Logic model for pilot evaluation.

**Inputs**	**Activities**	**Outputs**	**Short-term outcomes (Sept-Dec 2021)**	**Intermediate outcomes Jan-May 2022)**	**Long-term outcomes (2–3 years)**
– School teams – Project ECHO sessions – Toolkit – Program partners – Funding – Summit	– Participation in summit and ECHO sessions – Case development and reporting – Review of toolkit – Developing action plan – Implementing action plan – Learning from and collaborating with other school teams	– Action plan presentation – Lessons learned presentation	– Development of school teams. – Increased knowledge, skills, and self-efficacy among school team members. – Established network of school teams, stakeholders, and ECHO experts – Improved intra-school team coordination.	– Increased collaboration among school teams, stakeholders, and ECHO experts. – Increased access to vaping cessation resources. – Increased student referrals using the Ask Advise Connect (AAC) model. – Reduction in disciplinary action related to vaping among students. – Introduction of school policy/practice to address student vaping. – Implementation of school policy/practice comprehensively addressing vaping/nicotine dependence among students.	– Improved health behaviors (i.e., reduced vaping among students). – Improved health outcomes (i.e., reduced likelihood of long-term addiction; substance use; and respiratory health conditions). – School system changes due to established vaping policy/practice. – Consistent and evolving guidance around vaping cessation resources in schools.

The goals of the evaluation of the Vaping ECHO for Education pilot program are to:

- Assess pilot management and implementation.
- Assess level and quality of support provided to program participants by the Project ECHO team.
- Assess the extent to which the goals of the pilot program are being achieved.
- Assess how effectively school teams can develop an individualized action plan to address e-cigarette use.

The evaluation will include two components: *process* and *outcome*. The process evaluation component will assess (1) project management (how well the initiative is being managed and implemented); (2) technical assistance (the degree to which school teams have adequate support and resources to successfully implement the pilot); and (3) team-specific goals (the degree to which school teams can develop an action plan).

The outcome evaluation will assess (1) participant perceptions of capacity and skills to execute vaping cessation referrals and support for students in need; (2) the participant confidence in making policy, practice, and system changes that address e-cigarette use; and (3) school team referrals and actions specifically addressing e-cigarette use.

Evaluation outcomes were collected *via* self-report surveys and school action plans. The timing of surveys is depicted in Table 3. Evaluation question were set for each process and outcome component and will be addressed with a mix of quantitative and qualitative data, through descriptive analysis using mid-ECHO evaluations and pre-post comparisons.

**Table 3:** Assessment data source and timing.

**Instrument**	**Source of information**	**Timepoint**	**Evaluation component**
Pre-test survey	Self-report survey	Baseline (August 2021)	Outcome evaluation
Post-test survey	Self-report survey	Follow-up (April 2022)	Outcome evaluation
Component-specific survey	Self-report survey	Following each program component	Process evaluation
Action plan	School team action plans	Follow-up (January 2022)	Process evaluation
School team participation	Attendance record	Following each program component	Process evaluation

## Discussion

### Anticipated benefits

This pilot Vaping ECHO for Education aims to address a school-based need to address nicotine use in Kansas middle and high schools in order to maximize student health. We anticipate that, if successful, the pilot will result in both short- and long-term benefits to the schools, staff, and students. Specifically, in the short-term, it is anticipated that schools will build high functioning, skilled teams to address e-cigarette use. Teams will exhibit increased knowledge and competency, experience increased collaboration among school teams, greater referrals to cessation resources using evidence-based models such as Ask, Advise, Connect, and changes in policies to reduce punishment-based discipline for e-cigarette use. The following longer-term outcomes are anticipated two to three years following the pilot: (1) reductions in student vaping, (2) resultant improvements in student health, (3) system level changes in vaping-related policies and practice, and (4) cultural changes such that school or district norms do not support use of e-cigarettes.

### Unique features

The program included unique features that set it apart from many other ECHOs. First, the ECHO included a summit kick-off with nationally recognized speakers which functioned to align participants' goals and energize the school teams. Second, a toolkit of resources was provided ahead of each corresponding session and school teams were encouraged to review them and refer to them as they created their individualized plan for addressing e-cigarette use at their school. Third, component specific assessments were reviewed immediately following the sessions and allowed for real-time correction and response to learner feedback. Finally, schools created and submitted individualized action plans that were entirely school team-directed based on the provided training and were shared between school teams to encourage inter-team collaboration and work toward building a community of practice.

### Potential barriers or limitations

While we anticipate numerous benefits, the pilot is not without limitations. First, the ECHO ran during the second school year of the COVID-19 pandemic during two significant surges. As such, school teams' time was particularly limited and there were numerous competing priorities. It is unknown how this might impact the pilot outcomes. Second, the Vaping ECHO for Education was highly intensive and resulted in at least 12 h of in-session training and additional time spent devising and implementing action plans. While this resulted in a high-quality training experience, the time commitment may be seen as a barrier. Lastly, the program was piloted in 20 interdisciplinary schools across the state, each with unique approaches and implementation strategies, various degrees of access to resources, and each having different starting points in addressing vaping cessation in schools. It is unknown to what extent these differences will function as barriers to peer learning and collaboration.

### Dissemination plan

The findings from this pilot ECHO will be compiled by the evaluation team and published in peer-reviewed journals and presented at professional conferences. In addition, we will present methods and findings to state and regional tobacco control groups. Of note, we plan to include pilot participants in these presentations to ensure that the network of schools is represented in all dissemination efforts.

## Conclusions

If successful, the pilot findings would suggest that the ECHO model is an effective platform for disseminating evidence-based strategies to school staff. Moreover, the pilot will reach urban, rural, small, and large schools in the state of Kansas and, if successful, result in evidence-based policy changes and student and staff engagement in e-cigarette prevention and cessation. Moreover, the pilot could pave the way to implement the ECHO model to address other health-related issues faced by schools, providing access to high quality training in urban and rural areas of the state.

## Ethics statement

Ethical review and approval was not required for the study on human participants in accordance with the local legislation and institutional requirements. Written informed consent for participation was not required for this study in accordance with the national legislation and the institutional requirements.

## Author contributions

EL, JR, BF, MT, KD-C, and HS contributed to the conceptualization of the project, methods, and investigation. HS oversaw data analysis. KD-C served as project administrator. EL wrote the original draft of the paper. All authors provided critical feedback, reviewed, and approved the final manuscript.
