# Supplementary material for: Development and implementation of the ECHO model in a school setting to address youth electronic cigarette use in Kansas: A protocol
Source: Front Public Health. 2023 Jan 12;10:1057600. doi: 10.3389/fpubh.2022.1057600 (PMC9879567; doi:10.3389/fpubh.2022.1057600)
Supplement: Supplementary file 1 [file Data_Sheet_1.pdf]

Supplementary Figure 1. Pilot School Application

Kansas E-Cigarette/Vaping Project ECHO Pilot School Application

2021-2022 School Year

Overview

Thank you for your interest in the Kansas E-Cigarette/Vaping Project ECHO Pilot School opportunity. Here's some background on this initiative:

- In June 2019, the Kansas State Board of Education asked that a task force be formed to assist schools in dealing with E-Cigarette/Vaping use by students.
- The E-Cigarette/Vaping Task Force was formed and began working in July 2019.
- The Task Force has produced educational and informational materials and developed two documents that have been approved by the Kansas State Board of Education:
- Comprehensive Tobacco-Free School Policy
- Best Practices for Student Discipline for E-Cigarette/Vaping Policy Violations
- The Task Force has identified and shared information on quality web-based cessation resources.
- The Cessation Subgroup of the E-Cigarette/Vaping Task Force determined that a Vaping-focused Project ECHO would be instrumental in providing expertise and tools to help Kansas schools address vaping and the challenges posed by vaping.

Details about Pilot Schools:

- 20 Pilot Schools will be selected for participation in the Kansas Project ECHO on Vaping. 2 schools will be selected from each of the 10 Kansas State Board of Education member's districts.
- This is the first Project ECHO known to focus on vaping, so participating schools will serve as trailblazers.
- The selection of the Pilot Schools will be designed to reflect the variety and demographics of Kansas schools. The set of schools are anticipated to represent rural, urban, upper SES, and lower SES schools in addition to reflecting diversity and being geographically spread around the state.
- This Project ECHO is a coordinated effort between the Kansas State Department of Education (KSDE), the Kansas Department of Health and Environment (KDHE), the University of Kansas Medical Center, the Kansas Health Institute (KHI), and the University of Kansas Cancer Center.
- Participating Pilot Schools are expected to be motivated to impact student use of vaping products and committed to fully participating in the Project ECHO sessions.

Selection Considerations: (As noted earlier, the intent is for the selected schools to represent the following.)

- Geographic Representation
- Varied Size of Communities
- Diversity of Student Population
- Varied SES
- Commitment to Addressing Youth and Teen Vaping
- Team Composition and Approach

Timeline:

- Applications Released: May 18
- Completed Applications Due: June 15
- Pilot Schools Selected and Notified: June 29
- Pre-Summit Orientation: Aug. 11
- Summit: September 1
- ECHO Part 1: Sept. 15
- ECHO Part 2: Sept. 29
- ECHO Part 3: Oct. 13
- ECHO Part 4: Oct 27
- ECHO Part 5: Nov. 10
- School Team Presentations: Dec. 8
- Debriefing: Spring 2022

Benefits for Participating Schools:

- Participate in a ground-breaking approach to address vaping in schools.
- Create a school-centered team approach to address vaping in schools.
- Engage in a network of stakeholders committed to reducing vaping in schools.
- Increase confidence in schools' ability to address cessation needs of students.
- Develop participants' skills to provide support and referrals for vaping cessation.
- Utilize tele-mentoring to access expert advice concerning prevention and cessation.
- Apply the Whole School, Whole Community, Whole Child Model (WSCC) model, a student-centered framework developed by the Centers for Disease Control and Prevention. The WSCC calls for greater alignment between the public health and education sectors and emphasizes the importance of evidence-based school policies and practices.

Section A - General Information

District Number and Name (if applicable):

School Name:

County:

Key Applicant Contact: Title:

Mailing Address:

E-mail:

Telephone:

Additional Applicant Contact: Title:

E-mail:

Telephone:

Certification Signatures

We, the undersigned, certify that the information in this application is complete and we will support the applying school's participation as a Kansas E-Cigarette/Vaping Project ECHO pilot school.

School Contact Name:	School Contact Signature:	Date Signed:
Superintendent's Name:	Superintendent's Signature:	Date Signed:
Principal's Name:	Principal's Signature:	Date Signed:

Section B – School Demographics

Selection of Pilot Schools will not be based on any specific demographic qualifications. The intent is to have the Pilot Schools, collectively, reflect the greater population and variety of schools in Kansas.

2020-2021 School Enrollment:

Type of community: ☐ Urban ☐ Suburban ☐ Rural

Percentage of Free and/or Reduced Price Students:

State Board District:

Level of Vaping in Your School: ☐ Low ☐ Moderate ☐ High

Please complete the following two items:

Please provide a **brief** narrative describing your school's efforts to address student e-cigarette/vaping use as well as other tobacco products:

Pilot Schools are expected to have a "team" of people participate in the Project ECHO sessions. Each participating school will need to have a building-level administrator (i.e. Principal), and a health champion (i.e. School Nurse, School Counselor, Health Educator). Up to five individuals would be able to participate from each school. Indicate the participants below: *Required

- Building-level Administrator*:
- Health Champion*:
- Teacher:
- Athletic Trainer, Coach, Activity:
- Activity Sponsor:
- Community Member:
- Other (name and role):

Upload Saved Application by June 15 **HERE**. Please name files to include school and district before upload (e.g. ExampleShoolUSDxxx)

Office hours will be held on May 26 and June 2 from 4:30-5:00pm.

Zoom, follow this link **HERE**

If you cannot attend office hours, please send questions to the ECHO hub team. [email address]

Supplementary Figure 2. Action Plan Instructions and Template

Vaping ECHO for Education Action Plan

Pilot Period: January 2022-April 2022

Overview

The Action Plan (AP)* is a big picture plan of what will be needed to make progress in your school. This document is intended for school teams to fill out to plan work towards their vaping cessation outcomes. We encourage you to use this document for planning purposes and update it on ongoing basis. School teams will present their action plans during our December 8 session.

Goal

The goal of the AP is to support your success as you implement practice and policy change in your school. Specifically, the AP will provide an opportunity to regularly:

1) Identify and implement ongoing policy and practice efforts around your goal.
2) Learn from other schools about their efforts.
3) Adjust your vaping cessation strategies and capture lessons learned.

Timeframe:

Action Plan draft due date: December 8, 2021
Action Plan final due date: January 7, 2022

Tips for Filling Out Your Action Plan

- **Time Frame –** Each action plan has a specific time frame. Your action plan should be a reflection of the goals and work happening during this time period.
- **Previous Work –** If your school has worked in this area previously, please provide a short summary of that work. (For example, if you have started a referral program, have modified your curriculum, and so forth, that would be noted here.)
- **SMART Goals/Outputs –** These are the outputs you intend to measure during the specified time period and should be in SMART format. (Specific. Measurable. Achievable. Realistic. Timebound). You may add as many goals as you wish, we recommend 2-3 to start. *Goals and outputs may be eventually summarized in the Evaluation Plan.*
- **New Activities or Work** – List the major activities that lead to the accomplishment of the goal.
- **Policy change:** To be considered a policy, it needs to be 1) in writing, 2) be used to influence and guide decisions, 3) be passed by someone who has the authority to make those decisions, and 4) be at a governmental or organizational level. These policies range from a large-scale policy (e.g., district-wide referrals to community mental health centers) to smaller-scale policy (e.g., establishing a school team).
- **Practice changes:** To be considered a practice, it needs to be 1) in writing, 2) be used to influence and guide decisions, 3) be adopted by school practitioners, 4) be at the school level. These practices can range from a large-scale practice (e.g., changes to guidelines for disciplinary/rehabilitation action related to vaping) to a smaller-scale practice change (e.g., guidelines for open, student-centered conversations around vaping).

---

* *Vaping ECHO for Education adapted the action plan template developed by the Wichita State University Community Engagement Institute as part of the Pathways to a Healthy Kansas Initiative funded by Blue Cross and Blue Shield of Kansas, an independent licensee of the Blue Cross Blue Shield Association.*

Deeper Dive Questions

These questions are a quick guide to help school teams think deeper about their package planning activities.

**Vision:**<br>*If we come to a follow up meeting in April 2022 and feel pleased with the progress we have made compared with August 2021, what would we be doing differently? How often will we do it? With what populations will we implement these changes?*	
**Prior to first ECHO session:**<br>*What is our school struggling with to be successful with helping students stop vaping?*<br>*What do we need to learn to be successful?*<br>*If we learn how to talk to students about vaping, what would hold us back from using these skills with every student we know is vaping?*	
**Previous work:**<br>*What work has already been completed related vaping cessation?*<br>*What past events and/or items might be helpful for you to keep in mind related to vaping cessation?*<br>*What data does your school collect related to vaping cessation?*	
**Policy and/or Practice change:**<br>*How is the work impacting policy or practice in your school?*<br>*What specific impacts will be seen from implementing this action plan as it relates to policy and practice change?*<br>*Changes we will make with how we approach students include:*<br>*Changes we will make with our internal policies include:*<br>*Changes we will advocate for with our school board include:*	
**SMART Objectives:**<br>*What indicators would give you and your school the strongest sense of progress?*<br>*What is a realistic goal to meet related to your indicators?*<br>*What timeframe should be associated with this goal?*	
• *What steps do you have in mind to make progress on the objective above during the pilot period?*<br>• *What needs to happen during the pilot period to be able and make progress on the objective above?*	**(Insert Estimated Completion Dates)**

Vaping ECHO Action Plan

We suggest using *Table 1* before you attend your first ECHO session to help you plan your participation. The first set of questions are particularly helpful for planning. We suggest using *Table 2* to park any future ideas. We suggest using *Table 3a or 3b* to make your goals actionable.

Table 1. **Vision, Goals and Objectives to Improve Youth Access to Cessation Resources**

**Vision:**
**Prior to first ECHO session:**
**Previous work on vaping cessation:**
**High-level Goal(s):**

In Table 2, list goals or activities that you will not work on during this pilot but may be a possibility for your school in the future.

Table 2. **Future ideas**

Future Ideas	What needs would you be addressing future ideas?

Use Tables 3a or 3b (below) to flesh out your goals and objectives from Table 1. During this pilot period, you will be required to set at least:

- *two goals*
- *two tactics/objectives per goal*

Table 3b also provides an opportunity to capture lessons learned under "How did it work" section of the table that your team can discuss during the Spring 2022 session.

Table 3a. **Goals and Objectives, January 2022 to April 2022**

**SMART Goal:**	
**Policy and/or Practice Change:**	
**SMART Objective #1:**	
• **New activities or work**<br>•<br>•	**(Insert Estimated Completion Dates)**
**SMART Objective #2:**	
•<br>•<br>•	**(Insert Estimated Completion Date)**
**SMART Objective #3:**	
•<br>•<br>•	**(Insert Estimated Completion Date)**

Table 3b. **Goals and Tactics, January 2022 to April 2022**

Goal [2]	Audience	Tactic[3]	Responsible Party	Timeline	Needed Resource[4]	How did it Work?

[2] **Goal**: the end toward which effort is directed.
[3] **Tactic:** concrete step/action you can take to achieve your goal.
[4] **Needed resource**: an umbrella term that describes any tools, platforms, time, direct expenses associated with activities.
